# Berberine Sensitises Breast Cancer Cells to Radiation via the Attenuation of DNA Ligase III

**DOI:** 10.1111/jcmm.70836

**Published:** 2025-09-07

**Authors:** Yuxin Sun, Cong Li, Hang Yin, Kunyan Li, Ying Wang, Shuailong Zhang, Yi Zhao, Jing Wang, Weifeng Mao

**Affiliations:** ^1^ College of Basic Medical Sciences Dalian Medical University Dalian China; ^2^ Department of Oncology First Affiliated Hospital of Dalian Medical University Dalian China

**Keywords:** berberine, breast cancer, DNA damage repair, DNA ligase III, radiation

## Abstract

Berberine (BBR) is an isoquinoline alkaloid with a variety of biological activities, including anti‐microbial and anti‐tumoral activities. However, the cellular targets of BBR and the roles of BBR in the radiosensitivity of breast cancer cells are not well defined. In this study, we investigated the effects of BBR on the radiosensitivity of BT549 triple‐negative breast cancer cells. Through RNA‐seq results, we found that BBR significantly down‐regulated the expression of DNA ligases. The results of western blot and comet assay confirmed that BBR attenuated DNA ligase III (LIGIII) expression and caused DNA damage in a dose‐dependent manner. The results of electron microscopy showed that BBR decreased mitochondrial copies and induced mitochondrial autophagy. Clonal formation analysis showed that BBR sensitised breast cancer cells to irradiation. The genetic complementation and interference assays showed that the effect of BBR on the radiosensitivity of BT549 breast cancer cells is dependent on the expression of LIGIII. These results contribute to the understanding of the effects of BBR on cellular DNA repair and the clinical application of BBR in breast cancer therapy.

**Abbreviations**
BBR: berberine
HV1: Hipervariable region 1
IC50: 50% inhibitoryconcentration
LIGI: DNA ligase I
LIGIII: DNA ligase III
MTT: 3‐(4,5‐dimethylthiazol‐2‐yl)‐2,5‐diphenyltetrazolium bromide
TNBC: triple‐negative breast cancer cell

## 1 Introduction

Breast cancer is the most common malignant tumour with the lifetime risk about 10% for women, and causes approximately 685,000 deaths worldwide each year [1, 2, 3]. Triple‐negative breast cancer (TNBC), which is characterised by the absence of oestrogen receptor (ER), progesterone receptor (PR) and human epidermal growth factor receptor 2 (HER2), causes the highest risk of recurrence and the worst prognosis among all breast cancer subtypes [4, 5]. The standard therapy for breast cancer is surgery, followed by radiotherapy, chemotherapy or hormone therapy [6, 7, 8, 9, 10]. The efficacy of radiotherapy for breast cancer depends on the irradiation dose employed for patients; however, the high dose is limited to the range of safe applications, which also limits the efficacy of radiotherapy for breast cancer [11, 12]. It is necessary to seek an alternative to sensitise TNBC to irradiation to promote the efficacy of radiotherapy for breast cancer.

Berberine (BBR) is an alkaloid extracted from Coptis chinensis and Yellow cypress, which exhibits anti‐microbial and anti‐inflammation activities and is widely used in the treatments of metabolic and neurological problems [13, 14]. BBR also exhibits anti‐tumoral activities in different types of tumours [15, 16, 17, 18, 19, 20, 21, 22, 23]. In human oesophageal cancer cells and ovarian cancer cells, BBR down‐regulates the protein RAD51 and impairs DNA homologous recombination (HR) repair [18, 24]. BBR directly interacts with DNA to form a complex, induces DNA damage and sensitises breast cancer cells to chemotherapeutic and DNA damage agents including cisplatin, MMS and camptothecin, which suggest BBR impairs DNA repair [25, 26]. However, the targets of BBR on cellular DNA repair and the effects of BBR on radiosensitivity of breast cancer cells were not well defined.

Due to the critical roles of DNA repair in response to the damage caused by radiotherapy, targeting DNA repair is rational to improve the radiotherapeutic response. In cancer radiotherapy, HR repair and base excision repair (BER) are two major targeting pathways for the improvement of radiotherapy. Inhibition of Rad51, a key factor in HR, enhanced the radiation sensitivity of lung cancer [27]. XRCC1 is a critical factor in the BER pathway, which binds to DNA ligase III (LIGIII) and prevents the degradation of LIGIII [28]. The cancer cells with aberrant XRCC1 are more sensitive to radiation than the control cells [29]. As LIGIII is a key factor to seal the final nick of DNA damage in BER [30, 31], it is rational to improve radiotherapy by targeting LIGIII. However, due to the lack of a commercial LIGIII inhibitor, there have been rare reports about the effects of inhibition of LIGIII on cancer radiotherapy.

In this study, we investigated the effects of BBR on the sensitisation of TNBC cells to irradiation. We especially analyse the effects of BBR on the cellular expression of LIGIII, which helps to indicate the uniqueness of BBR's action on LIGIII compared to other radiosensitizers, such as Rad51 inhibitors in HR pathways. We analysed the effects of BBR on major DNA repair pathways including the HR repair pathway, non‐homologous end‐joining repair pathway, nucleotide excision repair pathway, mismatch repair pathway, BER pathway and the Fanconi anaemia repair pathway. The effects of BBR on apoptosis of tumour cells and chemotherapy have been reported, while its cellular targets and its roles in personalised medicine or combination cancer therapies were still not clear. Our findings contribute to understanding the actions of BBR in cellular DNA repair and the clinical employment of BBR in personalised medicine and combination therapies for breast cancer.

## 2 Materials

### 2.1 Cell Culture

The human breast cancer cell lines BT549 and human embryonic kidney 293T cells were obtained from American Type Culture Collection (ATCC, Manassas, USA). Cells were cultured in RPMI 1640 (Hyclone, Massachusetts, USA) and DMEM (Gibco, Massachusetts, USA) culture media supplemented with 10% heat‐inactivated fetal bovine serum (FBS, Hyclone, Massachusetts, USA), 100 mg/mL penicillin and 100 mg/mL streptomycin (Thermo Fisher Scientific, Massachusetts, USA).

### 2.2 Reagents and Antibodies

BBR was obtained from MCE (MedChemExpress, USA). Antibodies to β‐actin (HRP‐66009), LIGI (18051‐1‐AP) and LIGIII (26583‐1‐AP) were purchased from Proteintech.

### 2.3 MTT Assay

Cell viability was tested by MTT assay. 5 × 10^3^ cells were seeded in a well in 96‐well culture plates and cultured overnight. Then cells were incubated with various concentrations of BBR for 48 h. MTT was added to each well; after 4 h incubation, the cells were measured at 492 nm using a microplate photometer (Thermo Fisher Scientific, USA).

### 2.4 RNA Extraction

For BT549 cells and berberine‐treated BT549 cells, RNA was isolated using the Trizol‐chloroform method and quantified with NanoDrop‐2000.

### 2.5 RNA Sequencing Analysis

The RNA samples were sent to Shanghai Meiji Biomedical Technology Co. Ltd. mRNA was enriched with Oligo dT and fragmented, and the RNA fragments were reverse‐transcribed to create the final cDNA library. After quantification with TBS380, paired‐end sequencing was performed on Illumina Hiseq.

### 2.6 Western Blotting

Proteins were extracted using ice‐cold RIPA buffer with 1× protease inhibitors (Selleck, USA) and 1 mM PMSF (Sangon Inc., China). Lysates were separated by 10% SDS‐PAGE and transferred to PVDF membranes. Proteins were detected through various antibodies. Developments were analysed by gel imager (Biorad, USA) and the bands were analysed by Image Lab for statistical data.

### 2.7 Genomic DNA Extraction

Collected cells and added lysis buffer. Used RNase A and proteinase K to remove RNA and digest proteins. After that, DNA was purified by adding the same volume of a mixture of phenol: chloroform: isoamyl alcohol (25:24:1). After DNA was precipitated by anhydrous ethanol, TE buffer was added to dissolve the DNA. The DNA was quantified with NanoDrop‐2000. Hipervariable region 1 (HV1) and β‐actin were selected as the target fragments of mtDNA and nuclear DNA, respectively, which were detected by fluorescence quantitative RT‐qPCR experiments.

### 2.8 Reverse Transcription‐Quantitative PCR (RT‐qPCR)

After obtaining RNA samples, reverse transcription was performed using the One‐Step gDNA Removal and cDNA Synthesis SuperMix (Trans, AE311‐03). Preparation of suspensions was according to the TB Green Premix Ex Taq II (TaKaRa, RR820Q). Then the mix was performed in the Bioer 9600 FQD‐96A real‐time PCR detection system. Gene expression levels were normalised to β‐actin. A list of the primer sequences used for PCR or RT‐qPCR is provided in Table S1.

### 2.9 Comet Assay

Cells were treated with different doses of BBR for 48 h. 2 × 10^4^ cells were isolated for analysis. Dipped normal melting agarose into a frosted microscope slide, cells were mixed with 0.5% low melting agarose. The microscope slide was treated with ice‐cold lysis buffer for 2 h. Then the cells were denatured in alkaline buffer for 40 min and electrophoresed for 30 min at 25 V. The slides were washed three times by 0.4 mol/L Tris–HCl (pH 7.5), stained by 5 μg/mL ethidium bromide for 5 min. Cells were analysed in each slide using a fluorescence microscope. Scoring was performed using CometScore2.0 and CaspLab software to analyse the Tail DNA% and Tail lengths. Specific values for each parameter are detailed in the Table S6.

### 2.10 Transmission Electron Microscopy (TEM)

After BBR treatment for 48 h, cells were successively fixed with 4.5% glutaraldehyde for 2 h and osmium tetraoxide for 3 h. After washes with 0.1 M sodium pyrophosphate, the cell samples were dehydrated, polymerised at 70°C, cut into ultrathin sections, and stained with uranyl acetate and lead citrate. Images were observed and captured at the electron microscope.

### 2.11 Flow Cytometry for Apoptosis Detection

The experimental concentration of BBR was added while the BT549 cells were in the logarithmic growth phase. After 48 h of treatment, the cells were trypsinized and resuspended in 195 μl Annexin V‐binding buffer, then labelled with Annexin V‐FITC and PI for 20 min in the dark. The number of apoptotic cells was determined using flow cytometry.

### 2.12 Cell Growth Curves After Radiation Therapy

BT549 cells were plated, and when their confluence reached about 70%, BBR was added and incubated for 48 h. After changing to fresh medium, the cells were irradiated with different doses. After irradiation treatment, the cells were incubated for 6 h. Then, cells were collected, resuspended, counted and recorded to obtain the growth curve of cells after irradiation.

### 2.13 Clonal Formation Assay

BT549 cells were spread in six‐well plates at 500 cells per well and cultured for 14 days. 500 μL of 4% paraformaldehyde solution was added to each well and fixed at room temperature for 20 min. Then 500 μL of 0.1% crystal violet staining solution was added to each well at room temperature for 30 min. The crystal violet was aspirated, washed with ddH_2_O, and put into the oven for 1 h. After there were no water droplets, the cells were ready to be photographed.

### 2.14 Cloning and Lentiviral Transfection

Non‐targeting control and human LIGIII shRNA were cloned into pLKO.1 vector. Human LIGIIIM and LIGIIIN were cloned into pcDNA3.1 vector. The sequences for shRNAs used in this study are listed in Table S1. For shRNA knockdown lentiviral particles and overexpression of lentiviral particles were generated by transfecting 293T cells with 1.5 μg of ps‐PAX2, 0.5 μg of pMD4.G and 2 μg of targeted‐shRNA plasmids using Lipofectamine 2000 following the manufacturer's protocol. Viral supernatant was collected post 6 h of transfection. After the target cells were infected by the viral solution for 24 h, the medium was replaced with fresh medium and screened with puromycin for 1 week.

### 2.15 Statistical Analysis

Data was analysed by GraphPad Prism 7.0 software. Transcriptome sequencing data were analysed using the I‐Sanger platform. One‐way ANOVA was used for comparison among multiple groups; the *t*‐test method was used to compare two groups. *p < 0.05, **p < 0.01, ***p < 0.001 were considered statistically significant. “ns” means not significant. All experiments were performed in triplicate. Data are expressed as the mean ± standard deviation (SD).

## 3 Result

### 3.1 Berberine Decreased the Expression of LIGI and LIGIII, and Caused DNA Damages in BT549 Breast Cancer Cells

**FIGURE 1:**
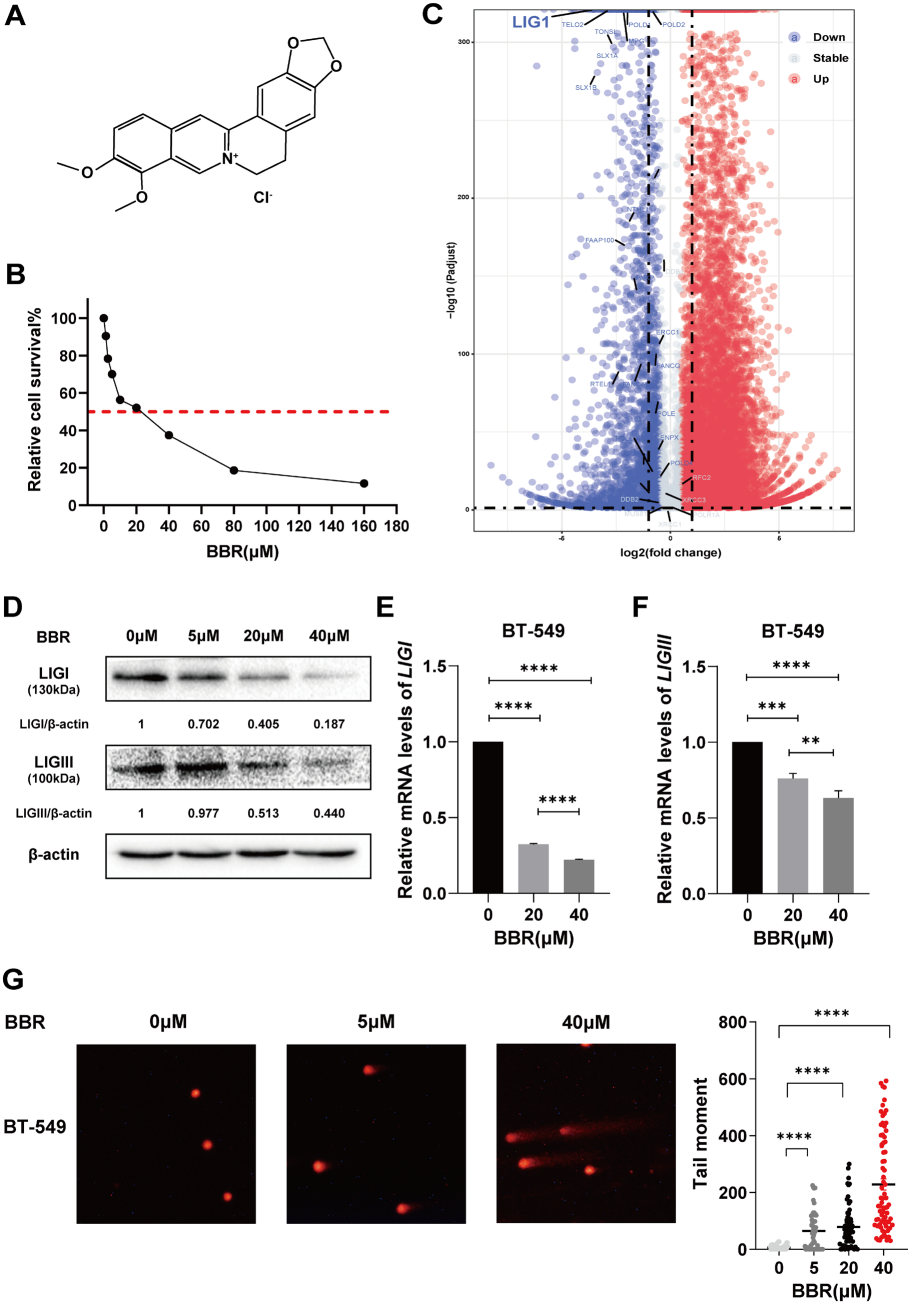
Berberine suppressed BT549 cell proliferation, decreased the expression of LIGI and LIGIII, and caused DNA damage. (A) The molecular structure of BBR. (B) Cell survival was detected by MTT assay. (C) Volcano diagram labelling the expressions of the DNA repair factors in BT549 cells treated with BBR. (D) Western blot was used to detect the expression of LIGI and LIGIII in BT549 cells after BBR treatment. Image Lab software was used to quantify the data. The ratio of LIGIII to β‐Actin was normalised. (E) RT‐qPCR assay was performed to detect the transcription of LIGI in BT549 cells treated by BBR, which showed the BBR decreased the transcripts of LIGI in a dose‐dependent manner. The bar graph shows mean ± SD (****p < 0.0001). (F) RT‐qPCR assay was performed to detect the transcription of LIGIII in BT549 cells treated by BBR, which showed the BBR decreased the transcripts of LIGIII in a dose‐dependent manner. The bar graph shows mean ± SD (**p < 0.01, ***p < 0.001, ****p < 0.0001). (G) Comet assay showed 0, 5 and 40 μM BBR promoted the tails of comet in the comet assay (left panel). The graph (right panel) showing quantification of the extent of DNA double‐strand breaks in BT549 cells. The tail lengths of cells were calculated and analysed by CaspLab software depicting mean ± SEM. *N* = 50 (**p < 0.01, ***p < 0.001).

BBR is an alkaloid, which structure is indicated in Figure 1A. We analysed the effects of BBR on BT549 cell proliferation by MTT assay. After 48 h treatment, BBR suppressed the growth of BT549 cells in a dose‐dependent manner. The IC_50_ value of BBR is about 20 μM (Figure 1B). We previously reported that BBR sensitised cancer cells to the DNA damage agent, cisplatin, and induced cellular DNA damage [23, 26]. To verify the effects of BBR on the expressions of genes related to DNA repair, we performed RNA‐seq in BT549 cells and berberine‐treated BT549 cells. We found that most of the genes related to the DNA damage repair pathway were up‐regulated (Figure S1A–G); however, DNA ligases were significantly down‐regulated (Figure S1A–G). As DNA ligases are the vital and final factors in DNA repair and the final enzymes to form the phosphodiester bond to seal the DNA nick in DNA repair, the roles of BBR in the down‐regulation of DNA ligases imply that BBR may render DNA damage unrepairable through inhibiting DNA ligases.

We next analysed the effects of BBR on the expressions of DNA ligases by western blot, which showed that BBR decreased the expressions of LIGI and LIGIII in a dose‐dependent manner (Figure 1D). We detected the effects of BBR on the transcription of LIGIII using RT‐qPCR assay, and found BBR decreased the transcriptions of LIGI and LIGIII (Figure 1E,F). As BBR down‐regulated LIGI and LIGIII, we detected the roles of BBR in DNA damage in BT549 cells using the comet assay, which showed BBR obviously increased the tail lengths, indicating BBR caused DNA damage in BT549 cells (Figure 1G).

### 3.2 Berberine Decreased Mitochondria Copies and Increased Autophagosomes in Mitochondria

**FIGURE 2:**
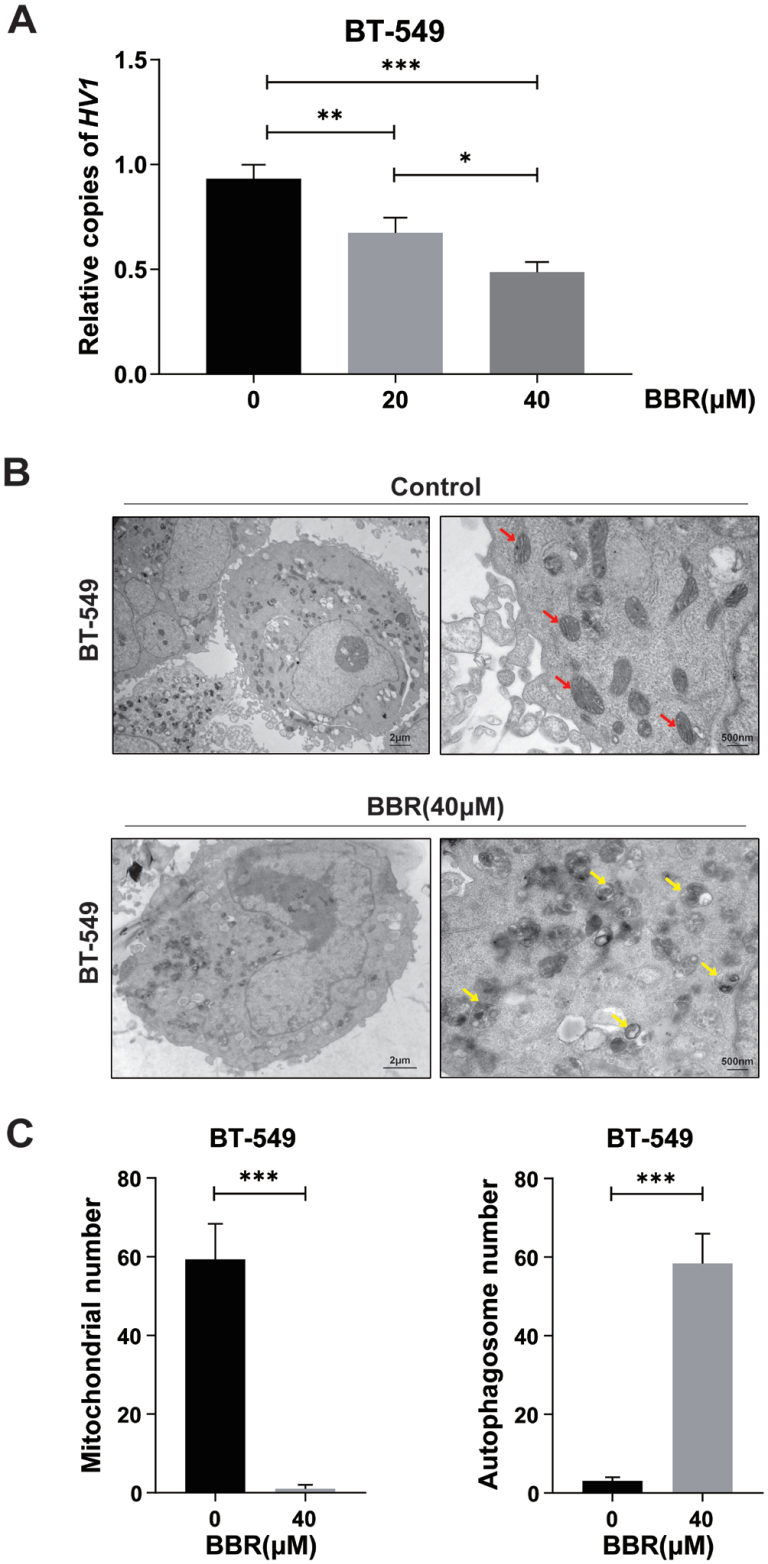
Berberine reduced mitochondria copies and induced more autophagosomes in mitochondria. (A) qPCR analysis of HV1 in BT549 cell lines with different concentrations of BBR, which reflects the copies of mitochondrial DNA in cells. Error bars denote mean ± SD (**p < 0.01, ***p < 0.001). (B) The numbers and morphology of mitochondria in BBR‐treated and control cells were observed through transmission electron microscopy, which showed BBR induced mitochondrial autophagosomes (yellow arrows) compared with mitochondria (red arrows) in control cells. (C) The numbers of mitochondria and autophagosomes were counted through transmission electron microscopy. Error bars denote mean ± SD (*p < 0.05, ***p < 0.001), *N* = 3.

LIGIII is the vital DNA ligase for mammalian mitochondria DNA repair and replication. After finding BBR decreased the expression of LIGIII, we further investigated whether BBR could affect mitochondrial DNA damage repair and DNA replication. We detected the mitochondrial copy numbers in BT549 cells through analysing mitochondrial HV1 and found that the mitochondrial copy numbers were reduced in BT549 cells treated with BBR (Figure 2A). Autophagosomes consist of a small portion of cytoplasm surrounded by a double‐layered membrane that can degrade its contents by fusing with lysosomes, which is a hallmark of mitochondrial autophagy. We also performed transmission electron microscopy experiments and found that BBR reduced mitochondrial copies and caused more autophagosomes in mitochondria (Figure 2B,C). Therefore, we concluded that BBR promoted mitochondrial autophagy in BT549 cancer cells and speculated that it was by inducing mitochondrial DNA damage. The RNA‐seq results showed that the transcripts of 12 core subunits of mitochondrial complex I were down‐regulated in BT549 cells treated with BBR (Figure S2A). Four of the core subunits were also detected by RT‐qPCR, and the results were consistent with the RNA‐seq results (Figure S2B). These results showed BBR interfered with mitochondrial replication.

### 3.3 Berberine Enhanced Radiosensitivity of BT549 Cells

**FIGURE 3:**
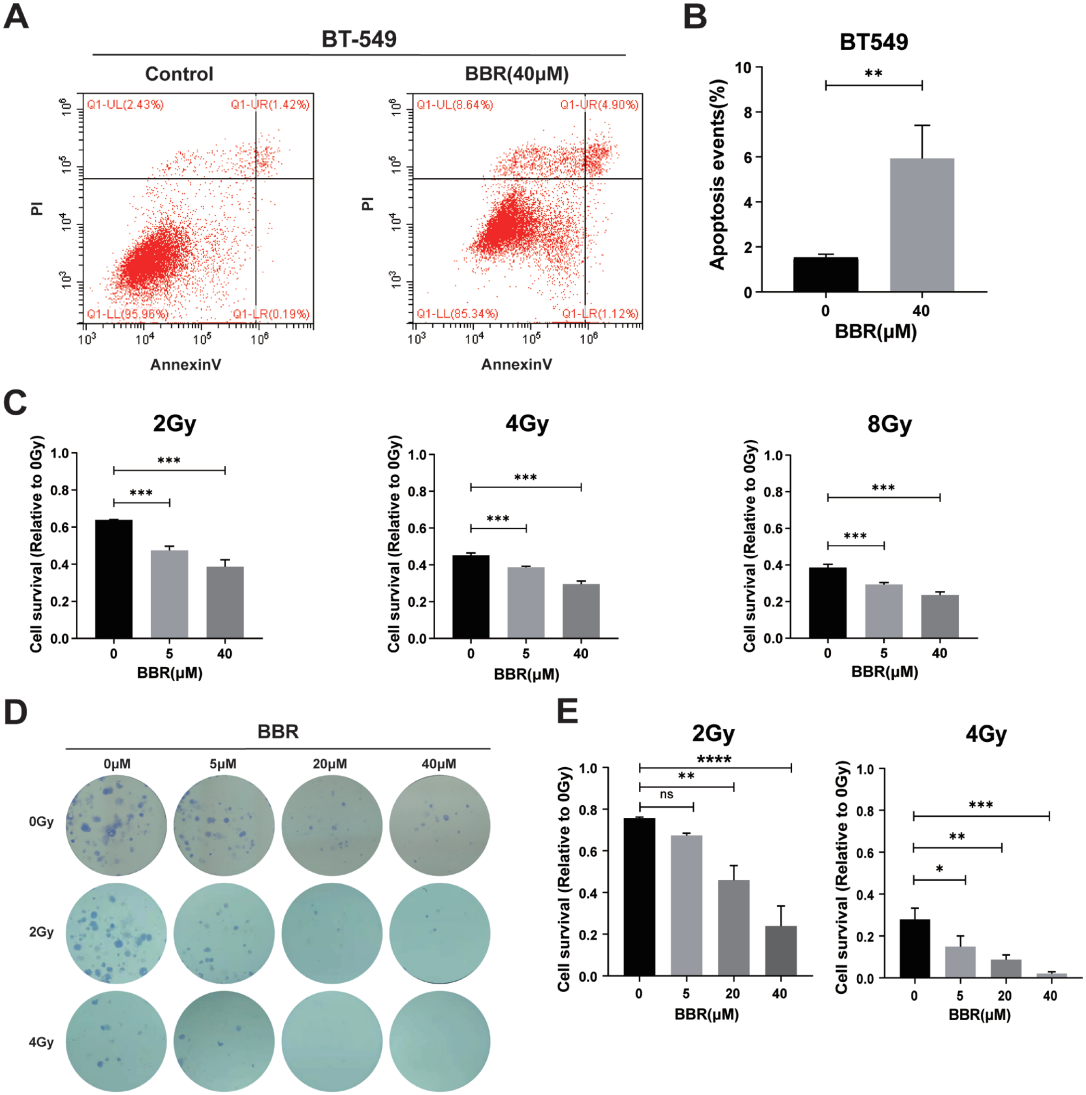
Berberine enhanced the radiosensitivity of BT549 cells. (A) The effects of BBR on apoptosis in BT549 cells were analysed by flow cytometry. (B) Flow cytometry results showed that BBR promoted apoptosis in BT549 cells. Error bars denote mean ± SD (**p < 0.01), *N* = 3. (C) Cells were counted and the cell numbers at 0 Gy of each BBR concentration were used as a control, and the ratios by comparing the cell numbers at 2 Gy and 4 Gy with the control were counted. Error bars denote mean ± SD (***p < 0.001), *N* = 3. (D) Clonal formation assay was used to detect the numbers of cell clone formations after radiation treatment. (E) The numbers of cell clones were counted. BBR enhanced the radiosensitivity at 2 Gy and 4 Gy through the clonal formation analysis. Error bars denote mean ± SD (***p < 0.001), *N* = 3.

We detected the effects of BBR on apoptosis of BT549 cells through flow cytometry, which showed that BBR promoted apoptosis of BT549 cells (Figure 3A,B). Based on the results that BBR inhibited LIGIII and caused DNA damage in BT549 cells, we detected the effects of BBR on radiosensitivity of BT549 cells. 2 Gy to 5 Gy doses of irradiation were used to investigate the radiosensitivity of MDA‐MB‐231 cancer cells and human ovarian cancer SKOV‐3 cancer cells [32, 33]. In clinical treatment, 2 to 10 Gy doses of irradiation were used for most cancer patients treating with low‐dose radiotherapy [34]. In this study, the BT549 cells were treated with BBR in combination with 2 Gy to 8 Gy doses of irradiation. The survivals of BT549 cells decreased after treating with BBR at different irradiation doses (Figure 3C). We also performed clonal formation assays in BT549 cells treated with BBR in combination with different doses of irradiation. The clonal formation abilities of BT549 cells decreased with the increasing doses of BBR and irradiation (Figure 3D,E). The results showed that 20 μM and 40 μM BBR obviously enhanced the radiosensitivity of BT549 cells at 2 Gy, and 5 μM BBR obviously increased the radiosensitivity of BT549 cells at 4 Gy.

### 3.4 Berberine Increased Radiosensitivity of BT549 Cells via the Attenuation of LIGIII

**FIGURE 4:**
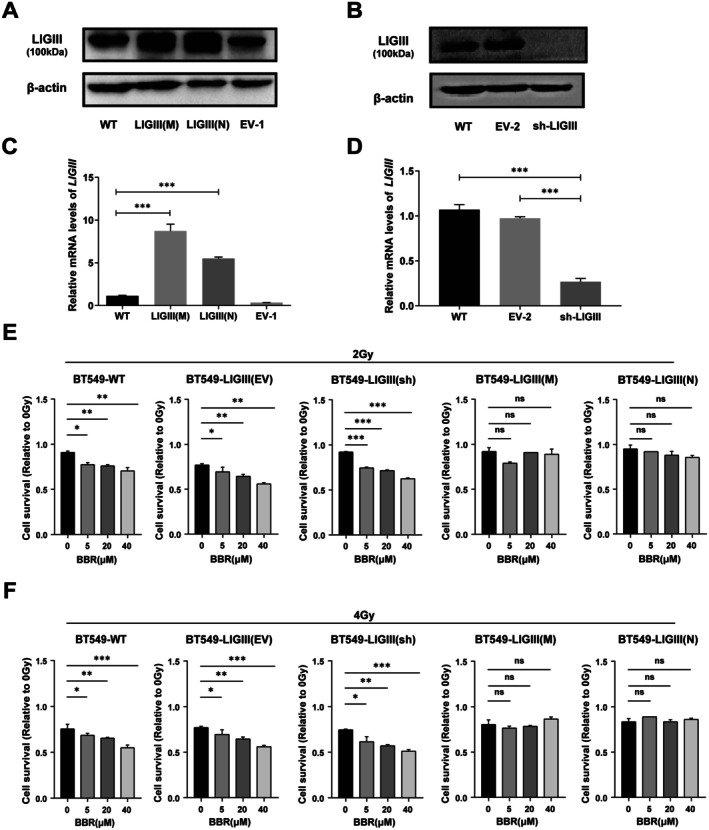
The effects of BBR on radiosensitivity are dependent on LIGIII. (A) The western blot results showed the expressions of LIGIII in BT549 cells. LIGIII(N) is the BT549 cells with over‐expressed nucleus LIGIII. LIGIII(M) is the BT549 cells with over‐expressed mitochondrial LIGIII. WT is the wildtype BT549 cells. EV‐1 is the BT549 cells with empty vector. (B) Western blot was used to detect LIGIII expression in WT, EV‐2(control vectors) and LIGIII knockdown (sh‐LIGIII) BT549 cells. (C, D) RT‐qPCR results showed LIGIII transcripts were up‐regulated in LIGIII(N) and LIGIII(M), and down‐regulated in LIGIII(sh) cells. Error bars denote mean ± SD (***p < 0.001), *N* = 3. (E, F) The over‐expressed LIGIII(N) and LIGIII(M) resisted the radiosensitivity caused by BBR at 2 Gy and 4 Gy in LIGIII(N) and LIGIII(M) cells. The cell numbers at 0 Gy of each BBR concentration treatment were used as a control. Error bars denote mean ± SD (ns, not significant; *p < 0.05, **p < 0.01, ***p < 0.001, ****p < 0.0001).

The above results suggested that BBR affected DNA damage repair and radiosensitivity through the inhibition of LIGIII. To confirm this hypothesis, we constructed PCDNA3.1‐LIGIIIN (nucleus) and PCDNA3.1‐LIGIIIM (mitochondria) plasmids, which expressed human nucleus LIGIII and mitochondrial LIGIII respectively. The BT549 cells stably over‐expressed nucleus LIGIII and mitochondria LIGIII were screened (Figure 4A,C). We also knocked down LIGIII in BT549 cells through RNA interference (Figure 4B,D). We treated the stably‐transfected cells with different concentrations of BBR for 48 h and performed irradiation. The results of the cell growth curve showed that the radiosensitivity of BT549 was increased in BT549 cells with the knockdown of LIGIII, while the overexpression of either nucleus LIGIII or mitochondrial LIGIII resisted the irradiation in BT549 cells treated with BBR (Figure 4E,F). Both nucleus LIGIII and mitochondria LIGIII possess the same core regions for the enzymatic activities. These results demonstrate that BBR enhances the radiosensitivity of BT549 cells through the attenuation of LIGIII.

## 4 Discussion

Radiotherapy is extensively used in the treatment of breast cancer patients; however, the employed doses of radiation need to be ranged in the safe radiation doses, which impedes the efficacy of radiotherapy of breast cancer [35], especially for TNBC patients processing the radioresistance and a high risk of recurrence [4, 5, 32]. We had reported BBR enhanced the chemo‐sensitivity of breast cancer cells [23, 26]. However, the cellular targets of BBR and the effects of BBR on the radiosensitivity of breast cancer were not clear. In this study, we treated BT549 cells with BBR and found that its IC_50_ was around 20 μM. Considering the heterogeneity among different tumour cells, we selected a wider range of BBR concentrations of 5, 20 and 40 μM to explore the radiosensitisation acts of BBR on BT549 cells. Through analysing the effects of BBR on the transcripts in the HR repair pathway [36], nucleotide excision repair pathway [37], mismatch repair pathway [38], BER pathway and the Fanconi anaemia repair pathway [39, 40], we found the transcripts of majorities of repair factors are up‐regulated; however, DNA ligases are obviously down‐regulated. As DNA ligase I and III are the vital and final factors in the single‐strand break repair to seal the DNA nick to form a phosphodiester bond [31, 41], the down‐regulation of DNA ligase I and III indicates the DNA repair was impaired by BBR.

The approaches for targeting DNA repair in cancer radiotherapy will have to confront several impediments, including tumour mutations that increase the DNA repair or the alternative DNA repair pathways that could compensate for a target or pathway inhibition, in this context with the inhibition of LIGIII. Three major DNA repair pathways are involved in the repair of DNA damage caused by radiation: HR, BER and non‐homologous end joining [42]. After LIGIII inhibition, tumour cells may alternatively choose HR and NHEJ pathways for DNA repair to resist radiotherapy; thus, the HR pathway would be targeted to generate a synthetic lethality effect. For example, targeting the BER pathway in combination with PARP1, a factor in the HR pathway, can cause synthetic lethality to inhibit tumour cell growth [43]. LIGIV and DNA‐PK are important repair factors in the NHEJ repair pathway. However, in tumour radiotherapy, targeting the NHEJ repair pathway is generally avoided. Although targeting DNA‐PK can enhance radiotherapy sensitivity [44], targeting the NHEJ repair still has side effects on normal tissues around the tumours. Also, radiotherapy theory relies on more mutations in DNA repair genes of tumour cells, which indicate HR and BER are more suitable repair pathways to target in radiotherapy. Our findings suggest that BBR has the potential to be used in combination with oncology chemotherapy drugs or targeted drugs such as PARP inhibitors to facilitate personalised and combination treatments for cancer. As LIGIII is the enzyme functioning in the repair of DNA breaks induced by radiation, we detected the effects of BBR on LIGIII in BT549 cells. Both RT‐qPCR and western blot results showed that BBR decreased the transcription and protein expression of LIGIII. The comet assay results showed BBR caused DNA damage in BT549 cells, suggesting BBR induced DNA breaks through interfering with LIGIII. LIGIII is the DNA ligase functioning in eukaryotic mitochondrial DNA repair and amplification. After we found BBR impaired LIGIII, we investigated the effects of BBR on mitochondrial amplification; the qPCR results showed BBR downregulated the mitochondrial copies. The electron transmission microscopy results showed that BBR increased the numbers of autophagosomes, suggesting BBR induced mitochondrial autophagy in breast cancer cells. BBR also increased the radiosensitivity of BT549 cells, while the BT549 cells with over‐expressed LIGIII resisted the radiosensitivity caused by BBR. These results demonstrate that BBR increases the radiosensitivity of breast cancer cells through the impairment of LIGIII.

In conclusion, we found BBR interfered with the majority of DNA repair pathways, and LIGIII is a cellular target of BBR. BBR sensitised breast cancer cells to radiation through the impairment of LIGIII, which would potentially contribute to increasing the efficacy of radiotherapeutic treatment of breast cancer. BBR reportedly down‐regulated the protein RAD51 and impaired DNA HR repair, which indicates the potential off‐target effects of BBR besides LIGIII on the cancer radiosensitivity. Whether BBR simultaneously inhibits other DNA ligases or has an antagonistic effect on other DNA ligases has not been well explored in this study. Also, in this study, only in vitro analysis was performed. A TNBC cell line, BT549, was employed to investigate the effects of BBR on breast cancer radiosensitivity. In vivo studies can help to better understand the potential effects of BBR across different types of cancers and the application of BBR in clinical cancer radiotherapy. For example, the assays employing tumour‐bearing mice and different doses of BBR combined with radiotherapy can help to explore the inhibitory effect of BBR on breast cancer. Overall, the potential clinical application of BBR in breast cancer chemotherapy combined with radiotherapy also needs to be further studied.

## Author Contributions

**Yuxin Sun:** data curation (equal), resources (equal), writing – original draft (equal). **Cong Li:** data curation (equal), investigation (equal), methodology (equal). **Kunyan Li:** data curation (supporting), investigation (supporting), methodology (supporting). **Ying Wang:** data curation (supporting). **Shuailong Zhang:** methodology (supporting). **Yi Zhao:** conceptualization (equal), funding acquisition (lead), investigation (equal), validation (supporting). **Weifeng Mao:** conceptualization (lead), funding acquisition (lead), writing – original draft (lead), writing – review and editing (lead). **Jing Wang:** conceptualization (equal), investigation (equal). **Hang Yin:** investigation (equal).

## Conflicts of Interest

The authors declare no conflicts of interest.

## Supporting information


**Figure S1:** The effects of BBR on gene expressions in DNA damage repairs. The effects of BBR on the transcripts in the base excision repair pathway (A), in the DNA replication repair pathway (B), in the Fanconi anaemia repair pathway (C), in the nucleotide excision repair pathway (D), in the non‐homologous end‐joining repair pathway (E), in the homologous recombination repair pathway (F), in the mismatch pathway (G).

**Figure S2:** BBR reduced the expressions of the core subunits of mitochondrial complex I. (A) Volcano diagram labelling the expressions of the core subunits of mitochondrial complex I in BT549 cells treated with BBR, in which most of complex I subunits were down‐regulated. (B) RT‐qPCR assay was performed to detect the transcripts of four core subunits of mitochondrial complex I, which showed the transcriptions of these subunits were decreased by BBR. Error bars denote mean ± SD (ns, not significant; *p < 0.05, **p < 0.01, ***p < 0.001, ****p < 0.0001), *N* = 3.

**Table S1:** qPCR primers and shRNA sequences used in the study.

**Table S2:** Cell survival (relative to 0 Gy) at 2 Gy irradiation intensity.
**Table S3:** Cell survival (relative to 0 Gy) at 4 Gy irradiation intensity.
**Table S4:** The grey value of western blot.
**Table S5:** The number of autophagosome or morphology.
**Table S6:** The dates for comet assay by CometScore2_0 (Three samples per group as an example).

## Data Availability

Data available on request from the authors.

## References

[1] M. Arnold , E. Morgan , H. Rumgay , et al., “Current and Future Burden of Breast Cancer: Global Statistics for 2020 and 2040,” Breast 66 (2022): 15–23, 10.1016/j.breast.2022.08.010. 36084384 PMC9465273

[2] F. Bray , A. Jemal , N. Grey , J. Ferlay , and D. Forman , “Global Cancer Transitions According to the Human Development Index (2008–2030): A Population‐Based Study,” Lancet Oncology 13, no. 8 (2012): 790–801, 10.1016/s1470-2045(12)70211-5. 22658655

[3] J. Ferlay , I. Soerjomataram , R. Dikshit , et al., “Cancer Incidence and Mortality Worldwide: Sources, Methods and Major Patterns in GLOBOCAN 2012,” International Journal of Cancer 136, no. 5 (2014): E359–E386, 10.1002/ijc.29210. 25220842

[4] Y. Li , H. Zhang , Y. Merkher , et al., “Recent Advances in Therapeutic Strategies for Triple‐Negative Breast Cancer,” Journal of Hematology & Oncology 15, no. 1 (2022): 121, 10.1186/s13045-022-01341-0. 36038913 PMC9422136

[5] R. Zhang , Y. Yang , W. Dong , et al., “D‐Mannose Facilitates Immunotherapy and Radiotherapy of Triple‐Negative Breast Cancer via Degradation of PD‐L1,” Proceedings of the National Academy of Sciences of the United States of America 119, no. 8 (2022): e2114851119, 10.1073/pnas.2114851119. 35181605 PMC8872783

[6] M. De Laurentiis , D. Cianniello , R. Caputo , et al., “Treatment of Triple Negative Breast Cancer (TNBC): Current Options and Future Perspectives,” Cancer Treatment Reviews 36 Suppl 3 (2010): S80–S86, 10.1016/s0305-7372(10)70025-6. 21129616

[7] B. Fisher , S. Anderson , J. Bryant , et al., “Twenty‐Year Follow‐Up of a Randomized Trial Comparing Total Mastectomy, Lumpectomy, and Lumpectomy Plus Irradiation for the Treatment of Invasive Breast Cancer,” New England Journal of Medicine 347, no. 16 (2002): 1233–1241, 10.1056/NEJMoa022152. 12393820

[8] O. Gluz , C. Liedtke , N. Gottschalk , L. Pusztai , U. Nitz , and N. Harbeck , “Triple‐Negative Breast Cancer—Current Status and Future Directions,” Annals of Oncology 20, no. 12 (2009): 1913–1927, 10.1093/annonc/mdp492. 19901010

[9] J. Kühnöl , C. Kühnöl , and D. Vordermark , “Radiotherapy of Brain Metastases From Breast Cancer: Treatment Results and Prognostic Factors,” Oncology Letters 11, no. 5 (2016): 3223–3227, 10.3892/ol.2016.4349. 27123095 PMC4840936

[10] F. Magnoni , G. Corso , P. Maisonneuve , et al., “A Propensity Score‐Matched Analysis of Breast‐Conserving Surgery Plus Whole‐Breast Irradiation Versus Mastectomy in Breast Cancer,” Journal of Cancer Research and Clinical Oncology 149, no. 3 (2023): 1085–1093, 10.1007/s00432-022-03973-8. 35254519 PMC11328321

[11] Y. Duan , T. Liu , Y. Zhou , T. Dou , and Q. Yang , “Glycoside Hydrolase Family 18 and 20 Enzymes Are Novel Targets of the Traditional Medicine Berberine,” Journal of Biological Chemistry 293, no. 40 (2018): 15429–15438, 10.1074/jbc.RA118.004351. 30135205 PMC6177593

[12] E. Shaw , C. Scott , L. Souhami , et al., “Single Dose Radiosurgical Treatment of Recurrent Previously Irradiated Primary Brain Tumors and Brain Metastases: Final Report of RTOG Protocol 90‐05,” International Journal of Radiation Oncology, Biology, Physics 47, no. 2 (2000): 291–298, 10.1016/s0360-3016(99)00507-6. 10802351

[13] A. Baska , K. Leis , and P. Gałązka , “Berberine in the Treatment of Diabetes Mellitus: A Review,” Endocrine, Metabolic & Immune Disorders Drug Targets 21, no. 8 (2021): 1379–1386, 10.2174/1568026620666201022144405. 33092516

[14] M. Imenshahidi and H. Hosseinzadeh , “Berberine and Barberry ( *Berberis vulgaris* ): A Clinical Review,” Phytotherapy Research 33, no. 3 (2019): 504–523, 10.1002/ptr.6252. 30637820

[15] Y. Kou , B. Tong , W. Wu , X. Liao , and M. Zhao , “Berberine Improves Chemo‐Sensitivity to Cisplatin by Enhancing Cell Apoptosis and Repressing PI3K/AKT/mTOR Signaling Pathway in Gastric Cancer,” Frontiers in Pharmacology 11 (2020): 616251, 10.3389/fphar.2020.616251. 33362566 PMC7756080

[16] G. Li , C. Zhang , W. Liang , Y. Zhang , Y. Shen , and X. Tian , “Berberine Regulates the Notch1/PTEN/PI3K/AKT/mTOR Pathway and Acts Synergistically With 17‐AAG and SAHA in SW480 Colon Cancer Cells,” Pharmaceutical Biology 59, no. 1 (2021): 21–30, 10.1080/13880209.2020.1865407. 33417512 PMC7808376

[17] Q. Tao , Q. Liu , H. Jiang , et al., “Berberine Radiosensitizes Human Esophageal Cancer Cells by Downregulating Homologous Recombination Repair Protein RAD51,” PLoS One 6, no. 8 (2011): e23427, 10.1371/journal.pone.0023427. 21858113 PMC3152570

[18] D. Hou , G. Xu , C. Zhang , et al., “Berberine Induces Oxidative DNA Damage and Impairs Homologous Recombination Repair in Ovarian Cancer Cells to Confer Increased Sensitivity to PARP Inhibition,” Cell Death & Disease 8, no. 10 (2017): e3070, 10.1038/cddis.2017.471. 28981112 PMC5680592

[19] S. M. Akula , S. Candido , M. Libra , et al., “Abilities of Berberine and Chemically Modified Berberines to Interact With Metformin and Inhibit Proliferation of Pancreatic Cancer Cells,” Advances in Biological Regulation 73 (2019): 100633, 10.1016/j.jbior.2019.04.003. 31047842

[20] J. A. McCubrey , S. L. Abrams , K. Lertpiriyapong , et al., “Effects of Berberine, Curcumin, Resveratrol Alone and in Combination With Chemotherapeutic Drugs and Signal Transduction Inhibitors on Cancer Cells—Power of Nutraceuticals,” Advances in Biological Regulation 67 (2018): 190–211, 10.1016/j.jbior.2017.09.012. 28988970

[21] Y. Pan , D. Shao , Y. Zhao , et al., “Berberine Reverses Hypoxia‐Induced Chemoresistance in Breast Cancer Through the Inhibition of AMPK‐HIF‐1α,” International Journal of Biological Sciences 13, no. 6 (2017): 794–803, 10.7150/ijbs.18969. 28656004 PMC5485634

[22] J. Du , Y. Sun , Y. Y. Lu , et al., “Berberine and Evodiamine Act Synergistically Against Human Breast Cancer MCF‐7 Cells by Inducing Cell Cycle Arrest and Apoptosis,” Anticancer Research 37, no. 11 (2017): 6141–6151, 10.21873/anticanres.12063. 29061795

[23] Y. Zhao , Z. Jing , Y. Li , and W. Mao , “Berberine in Combination With Cisplatin Suppresses Breast Cancer Cell Growth Through Induction of DNA Breaks and Caspase‐3‐Dependent Apoptosis,” Oncology Reports 36, no. 1 (2016): 567–572, 10.3892/or.2016.4785. 27177238

[24] Q. Liu , H. Jiang , Z. Liu , et al., “Berberine Radiosensitizes Human Esophageal Cancer Cells by Downregulating Homologous Recombination Repair Protein RAD51,” PLoS One 6, no. 8 (2011): e23427, 10.1371/journal.pone.0023427. 21858113 PMC3152570

[25] Z. Yin , E. Chen , X. Cai , et al., “Baicalin Attenuates XRCC1‐Mediated DNA Repair to Enhance the Sensitivity of Lung Cancer Cells to Cisplatin,” Journal of Receptor and Signal Transduction Research 42, no. 3 (2022): 215–224, 10.1080/10799893.2021.1892132. 33719846

[26] X. Gao , J. Wang , M. Li , et al., “Berberine Attenuates XRCC1‐Mediated Base Excision Repair and Sensitizes Breast Cancer Cells to the Chemotherapeutic Drugs,” Journal of Cellular and Molecular Medicine 23, no. 10 (2019): 6797–6804, 10.1111/jcmm.14560. 31338966 PMC6787507

[27] A. Sak , G. Stueben , M. Groneberg , W. Böcker , and M. Stuschke , “Targeting of Rad51‐Dependent Homologous Recombination: Implications for the Radiation Sensitivity of Human Lung Cancer Cell Lines,” British Journal of Cancer 92, no. 6 (2005): 1089–1097, 10.1038/sj.bjc.6602457. 15785736 PMC2361929

[28] M. L. Hegde , T. K. Hazra , and S. Mitra , “Early Steps in the DNA Base Excision/Single‐Strand Interruption Repair Pathway in Mammalian Cells,” Cell Research 18, no. 1 (2008): 27–47, 10.1038/cr.2008.8. 18166975 PMC2692221

[29] J. Thacker and M. Z. Zdzienicka , “The Mammalian XRCC Genes: Their Roles in DNA Repair and Genetic Stability,” DNA Repair 2, no. 6 (2003): 655–672, 10.1016/s1568-7864(03)00062-4. 12767346

[30] A. E. Tomkinson and A. Sallmyr , “Structure and Function of the DNA Ligases Encoded by the Mammalian LIG3 Gene,” Gene 531, no. 2 (2013): 150–157, 10.1016/j.gene.2013.08.061. 24013086 PMC3881560

[31] A. Sallmyr , S. K. Bhandari , T. Naila , and A. E. Tomkinson , “Mammalian DNA Ligases; Roles in Maintaining Genome Integrity,” Journal of Molecular Biology 436, no. 1 (2024): 168276, 10.1016/j.jmb.2023.168276. 37714297 PMC10843057

[32] Y. H. Su , Y. Z. Wu , D. K. Ann , J. L. Chen , and C. Y. Kuo , “Obesity Promotes Radioresistance Through SERPINE1‐Mediated Aggressiveness and DNA Repair of Triple‐Negative Breast Cancer,” Cell Death & Disease 14, no. 1 (2023): 53, 10.1038/s41419-023-05576-8. 36681663 PMC9867751

[33] M. S. Aleissa , M. Al‐Zharani , L. M. Alneghery , and A. M. Aleissa , “Berberine Enhances the Sensitivity of Radiotherapy in Ovarian Cancer Cell Line (SKOV‐3),” Saudi Pharmaceutical Journal: SPJ: The Official Publication of the Saudi Pharmaceutical Society 31, no. 1 (2023): 110–118, 10.1016/j.jsps.2022.11.009. 36685297 PMC9845113

[34] H. Menon , D. Chen , R. Ramapriyan , et al., “Influence of Low‐Dose Radiation on Abscopal Responses in Patients Receiving High‐Dose Radiation and Immunotherapy,” Journal for Immunotherapy of Cancer 7, no. 1 (2019): 237, 10.1186/s40425-019-0718-6. 31484556 PMC6727581

[35] D. H. Roukos , “Radiation Therapy for Breast Cancer,” New England Journal of Medicine 360, no. 13 (2009): 1362; author reply 1363. 19330940

[36] J. Li , H. Sun , Y. Huang , Y. Wang , Y. Liu , and X. Chen , “Pathways and Assays for DNA Double‐Strand Break Repair by Homologous Recombination,” Acta Biochimica et Biophysica Sinica 51, no. 9 (2019): 879–889, 10.1093/abbs/gmz076. 31294447

[37] J. A. Marteijn , H. Lans , W. Vermeulen , and J. H. Hoeijmakers , “Understanding Nucleotide Excision Repair and Its Roles in Cancer and Ageing,” Nature Reviews Molecular Cell Biology 15, no. 7 (2014): 465–481, 10.1038/nrm3822. 24954209

[38] R. Fishel , “Mismatch Repair,” Journal of Biological Chemistry 290, no. 44 (2015): 26395–26403, 10.1074/jbc.R115.660142. 26354434 PMC4646297

[39] J. D. Peake and E. Noguchi , “Fanconi Anemia: Current Insights Regarding Epidemiology, Cancer, and DNA Repair,” Human Genetics 141, no. 12 (2022): 1811–1836, 10.1007/s00439-022-02462-9. 35596788

[40] E. E. Kennedy , P. J. Caffrey , and S. Delaney , “Initiating Base Excision Repair in Chromatin,” DNA Repair 71 (2018): 87–92, 10.1016/j.dnarep.2018.08.011. 30170831 PMC6340775

[41] H. B. Lieberman , “DNA Damage Repair and Response Proteins as Targets for Cancer Therapy,” Current Medicinal Chemistry 15, no. 4 (2008): 360–367, 10.2174/092986708783497328. 18288990

[42] W. L. Santivasi and F. Xia , “Ionizing Radiation‐Induced DNA Damage, Response, and Repair,” Antioxidants & Redox Signaling 21, no. 2 (2014): 251–259, 10.1089/ars.2013.5668. 24180216

[43] A. Ray Chaudhuri and A. Nussenzweig , “The Multifaceted Roles of PARP1 in DNA Repair and Chromatin Remodelling,” Nature Reviews Molecular Cell Biology 18, no. 10 (2017): 610–621, 10.1038/nrm.2017.53. 28676700 PMC6591728

[44] K. Nakamura , A. Karmokar , P. M. Farrington , et al., “Inhibition of DNA‐PK With AZD7648 Sensitizes Tumor Cells to Radiotherapy and Induces Type I IFN‐Dependent Durable Tumor Control,” Clinical Cancer Research: An Official Journal of the American Association for Cancer Research 27, no. 15 (2021): 4353–4366, 10.1158/1078-0432.Ccr-20-3701. 34011558 PMC9401489

