# Supplementary figures and images for: Berberine Sensitises Breast Cancer Cells to Radiation via the Attenuation of DNA Ligase III
Source: J Cell Mol Med. 2025 Sep 7;29(17):e70836. doi: 10.1111/jcmm.70836 (PMC12414796; doi:10.1111/jcmm.70836)

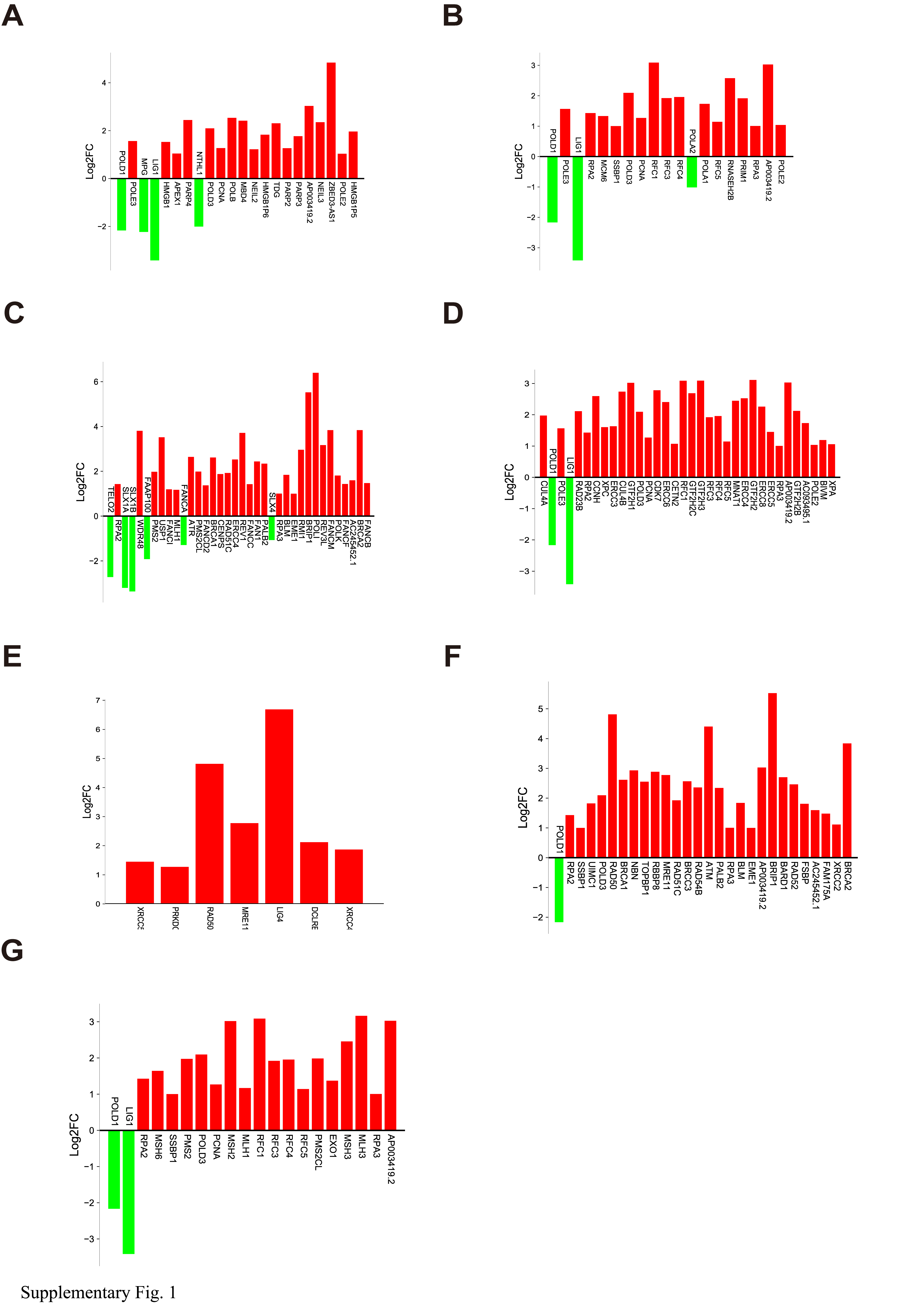

Supplement: Supplementary file 1 — Figure S1: The effects of BBR on gene expressions in DNA damage repairs. The effects of BBR on the transcripts in the base excision repair pathway (A), in the DNA replication repair pathway (B), in the Fanconi anaemia repair pathway (C), in the nucleotide excision repair pathway (D), in the non‐homologous end‐joining repair pathway (E), in the homologous recombination repair pathway (F), in the mismatch pathway (G). [file JCMM-29-e70836-s002.tif]

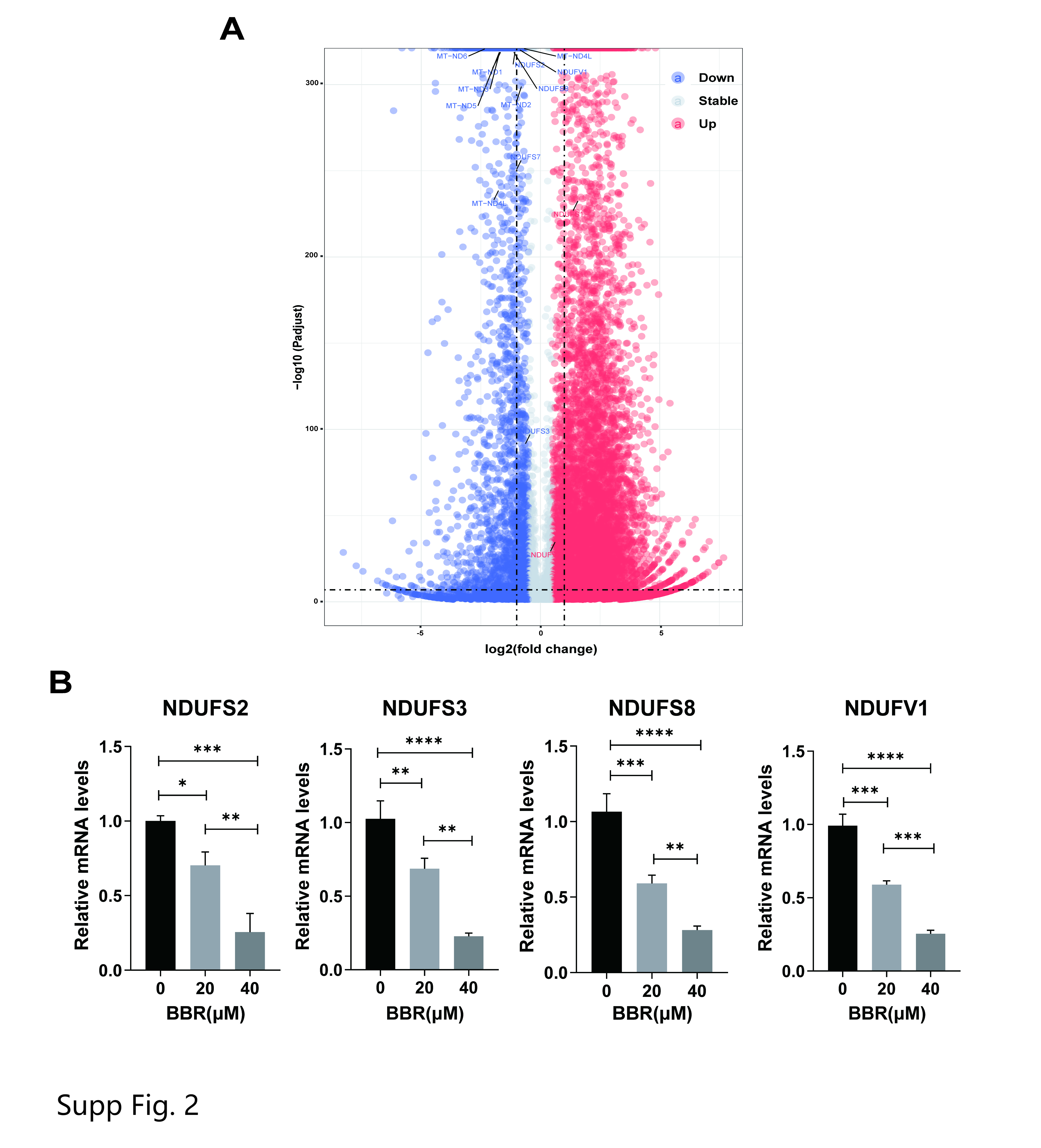

Supplement: Supplementary file 2 — Figure S2: BBR reduced the expressions of the core subunits of mitochondrial complex I. (A) Volcano diagram labelling the expressions of the core subunits of mitochondrial complex I in BT549 cells treated with BBR, in which most of complex I subunits were down‐regulated. (B) RT‐qPCR assay was performed to detect the transcripts of four core subunits of mitochondrial complex I, which showed the transcriptions of these subunits were decreased by BBR. Error bars denote mean ± SD (ns, not significant; *p < 0.05, **p < 0.01, ***p < 0.001, ****p < 0.0001), N = 3. [file JCMM-29-e70836-s001.tif]

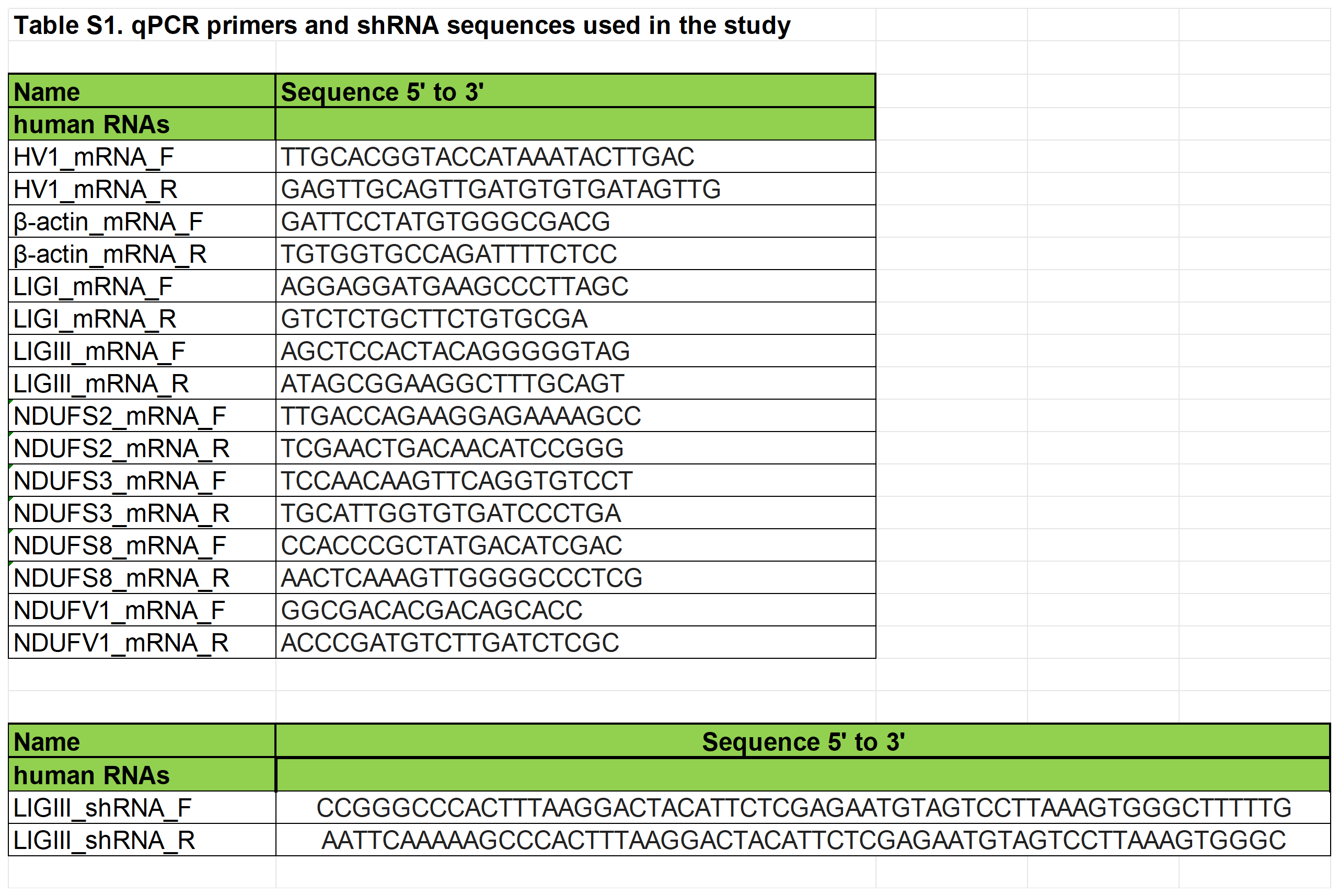

Supplement: Supplementary file 3 — Table S1: qPCR primers and shRNA sequences used in the study. [file JCMM-29-e70836-s003.png]
